# Assessing the feasibility, usability and acceptability of the *MySafeRx* platform among individuals in outpatient buprenorphine treatment: Lessons learned from a pilot randomized controlled trial

**DOI:** 10.1016/j.dadr.2022.100045

**Published:** 2022-03-17

**Authors:** Grace E. Janzow, Cassandra Harding, Michael Flores, Jacob Borodovsky, Jackson Steinkamp, Lisa A. Marsch, Zev Schuman-Olivier

**Affiliations:** a Cambridge Health Alliance, Harvard Medical School, Department of Psychiatry, 1035 Cambridge Street, Suite 21A – CMC, Cambridge, MA 02141, United States; b Center for Technology and Behavioral Health, Geisel School of Medicine at Dartmouth College, EverGreen Center, Suite 315, NH 03766, Lebanon

**Keywords:** Buprenorphine, Telehealth, Coaching, Recovery, Opioid use, Motivational interviewing

## Abstract

**Background:**

Increasing buprenorphine/naloxone (B/N) access for opioid use disorder (OUD) is essential yet ensuring adherence and preventing diversion remains challenging. This study examines the feasibility, usability, and acceptability of *MySafeRx*, a mobile platform integrating motivational coaching, adherence monitoring, and electronic dispensing during office-based B/N treatment.

**Methods:**

In this multi-site randomized controlled trial, *MySafeRx* provided coaching and supervised self-administration of B/N by mobile recovery coaches (MRCs) via videoconference. Referred adults (ages 18–65) with OUD were randomized to 1) 42-days of adjunctive *MySafeRx* treatment (*n* = 13) or 2) a standard care control group (*n* = 14).

**Results:**

The randomized sample was 63% female and 100% White. Twelve of 13 *MySafeRx* participants completed at least one MRC session. The mean system usability score reported by *MySafeRx* participants was 78.4 (*n* = 12). Participants indicated they would recommend *MySafeRx* to a friend (mean= 4.1 of 5), and that the dispenser (4.1 of 5) and videoconferencing (4.2 of 5) were easy to use. The MRC component had the highest acceptability (4.4 of 5). MRCs observed B/N self-administration for an average of 64.3% of the required study days (men: 68.9%; women: 57.9%). On average, men (*n* = 4) met with MRCs on 32±14 days versus 47±6 days for women (*n* = 8). Exploratory analyses did not show significant differences between intervention and control groups.

**Conclusions:**

Despite the small sample, this study supports usability and acceptability of *MySafeRx*. Increased adherence monitoring, even with remote coaching had limited appeal, which impacted feasibility due to slow recruitment, especially as community prescribing with relaxed monitoring requirements became more widespread.

## Highlights

• Motivational recovery coaching was well-received during periods of instability.
• *MySafeRx* intervention participants reported high levels of acceptability.
• Despite high platform acceptability, adherence monitoring did not have broad appeal.
• Recruitment was increasingly difficult as B/N prescribing became more widespread.

## 1 Introduction

Opioid Use Disorder (OUD) remains a public health crisis in the United States (Lyden and Binswanger, 2019). In the first quarter of 2020 alone (prior to national lockdowns for the COVID-19 pandemic), more than 19,000 individuals died from drug overdose in the United States – a 16% increase relative to the same time period in 2019 (Stephenson, 2020). Overdose deaths have only continued to increase in response to the pandemic, as access to clinical support for OUD has become increasingly limited and individuals more isolated (Henry et al., 2020; Khatri and Perrone, 2020; Krawczyk et al., 2020; Uscher-Pines et al., 2020).

Office-Based Buprenorphine Treatment (OBOT) for OUD is an effective and safe means of treating OUD and reducing overdose deaths (LaBelle et al., 2016; Larochelle et al., 2018). Evidence-based opioid agonist treatments including buprenorphine/naloxone (B/N) and methadone treatment remain primary clinical treatment methods (LaBelle et al., 2016). Despite the efficacy of B/N treatment in reducing relapse and overdose risk, medication non-adherence persists among various OUD populations (DiMatteo et al., 2007; Fareed et al., 2014; Lofwall and Walsh, 2014; Schuman-Olivier et al., 2018; Warden et al., 2012). Multiple factors contribute to non-adherence or partial adherence in the OUD population prescribed B/N. These factors include 1) ambivalence (e.g., related to abstaining from opioids, taking B/N daily, and being in addiction treatment), 2) psychiatric comorbidities, 3) the experience of shame or stigma around taking B/N, and 4) logistical issues (e.g., living and family environment, transportation, and lack of financial means to maintain recovery) (Arnett, 2000; Dayal and Balhara, 2017; DiMatteo et al., 2007; Fareed et al., 2014; Hadland et al., 2018; Rosenblum et al., 2011; Schuman-Olivier et al., 2014; Sussman and Arnett, 2014).

Common systemic issues existing prior to the COVID-19 pandemic such as disconnect between patient needs and provider availability, insufficient monitoring of medication adherence or toxicology screening, lack of accessible psychosocial treatment options, and the growing black market for diverted buprenorphine, each have complicated patient recovery and contributed to medication non-adherence or partial adherence (Accurso and Rastegar, 2016; Lin and Knudsen, 2019; Lofwall and Walsh, 2014). In the wake of the pandemic, added strains to the U.S. healthcare system and heightened financial and health-related stressors for individuals have only exacerbated the severity of these factors on patient success in addiction recovery (Davis and Samuels, 2020; Henry et al., 2020; Khatri and Perrone, 2020; Leppla and Gross, 2020; Slavova et al., 2020; Uscher-Pines et al., 2020).

*MySafeRx* is a technology-based mobile intervention integrating daily remote medication self-administration and motivational mobile recovery coaching. The platform was established prior to the COVID-19 pandemic to address the complex factors impacting OUD medication-adherence and access to OBOT treatment (Schuman-Olivier et al., 2018). *MySafeRx* promotes remote daily B/N adherence and reliable clinical support to reduce logistic burdens and enhance OUD treatment and recovery outcomes (Schuman-Olivier et al., 2018). The platform includes a combination of remote daily check-ins with mobile recovery coaches (MRCs) trained in motivational interviewing (MI), text-messaging reminders, secure storage of B/N medication through a secure electronic pill dispenser, and a standardized protocol for supervising self-administration of medication by *MySafeRx* MRCs via videoconference (Schuman-Olivier et al., 2018).

Building on a proof-of-concept pilot study conducted from 2016 to 2018 (Schuman-Olivier et al., 2018), the multi-site pilot randomized controlled trial (RCT) presented here assessed the feasibility, usability, and acceptability of the *MySafeRx* platform. This paper describes lessons learned from implementing the *MySafeRx* study protocol in various OBOT settings with varied contextual factors, including differing participant, gender, and community attitudes towards project logistics and implementation.

## 2 Methods

### 2.1 Study design

This was a multi-site, pilot RCT comparing *MySafeRx* to standard care during OBOT with B/N. Recruitment included participants ages 18–65 inclusive, across two clinical OBOT networks: the State of Vermont Blueprint for Health ‘Hub and Spoke’ network in Bennington, Vermont (Brooklyn and Sigmon, 2017) and the Cambridge Health Alliance (CHA) OBOT network in the metro-North Boston region in Massachusetts. Three primary study aims were registered (ClinicalTrials.gov Identifier: NCT02778282): Demonstrate (1) feasibility of *MySafeRx,* by participants achieving supervised self-administration of B/N on > 5/7 days per week for at least two-thirds of participants randomized to the study intervention arm, with no reports of substantial B/N diversion; (2) system usability, through a mean usability score of greater than 68, the a priori threshold of adequate usability on a well-validated System Usability Scale composed of 10 items on a 5-point Likert scale from “Strongly Disagree” to “Strongly Agree,” (Peres et al., 2013); and (3) acceptability, through a mean overall participant satisfaction score of greater than 3 of 5 on a satisfaction scale ranging from “Strongly Disagree” (score of 1) to “Strongly Agree” (Score of 5) at the conclusion of the study intervention period (42 days).

### 2.2 Participant recruitment and screening

Participants were recruited through referral from B/N prescribers or OBOT nurse care managers in the Vermont ‘Hub and Spoke,’ with referrals coming from the Bennington, Vermont, and through Cambridge Health Alliance (CHA) in Somerville, Massachusetts (Brooklyn and Sigmon, 2017, LaBelle et al., 2016, Schuman-Olivier et al., 2014). Study posters additionally publicized recruitment in public spaces at these specified clinics (e.g.,swaiting rooms). Recruitment occurred between March 2017 and March 2020. While OBOT clinical staff referred prospective participants to the study, they retained primary responsibility for their treatment. All referred participants completed an informed consent process approved by the Dartmouth and CHA Institutional Review Boards. After consenting, participants then completed screening, including demographics, psychiatric history, assessment of comfort with technology, and the Montreal Cognitive Assessment (Nasreddine et al., 2005).

Eligible participants were: (1) ages 18–65; (2) able to provide verbal and written informed consent; (3) in prescribed buprenorphine treatment for clinically diagnosed opioid use disorder (DSM-5, confirmed by referral providers through ICD-10 codes); (4) able to meet in a safe place for scheduled daily videoconferencing; and (5) had a positive opioid urine toxicology for non-prescribed or illicit opioids in the past month or had a missed urine toxicology with a reported admission of illicit opioid use in the past 30 days while in treatment for opioid use. Providers were encouraged to refer adults with recent use or missed urine screens. Most participants were either at risk of termination from OBOT treatment for ongoing illicit opioid use or were newly started on B/N and were still using opioids or felt to be at high risk of opioid relapse by their treatment providers.

Participants were excluded if they: (1) did not speak English or were not able to read the informed consent; (2) were in their third trimester of pregnancy; (3) had cognitive deficits with determined limited ability to complete study procedures (Montreal Cognitive Assessment score < 25/30) (Nasreddine et al., 2005); (4) homeless; (5) had a DSM-IV diagnosis of dementia, neurodegenerative disease, or other organic mental disorder, mental retardation, or autism; (6) were actively homicidal or suicidal with an imminent plan; (7) had a serious unstable medical illness including: cardiovascular, hepatic, renal, respiratory, endocrine, neurological, or hematological disease such that hospitalization for treatment of that illness was likely, an abnormal cardiovascular event, or uncontrolled hypertension in the past 60 days; (8) had used investigational medication in past 30 days. Homeless individuals were excluded due to safety and privacy concerns pertaining to safe device storage, as well as refusal by several local shelters to allow access to the device. However, those who were minimally housed, unstably housed, or fleeing domestic violence were all eligible for study participation.

### 2.3 Intervention

**Fig. 1:**
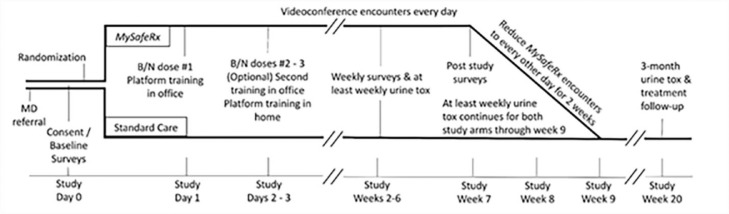
*MySafeRx* intervention study design. Participants randomized to the *MySafeRx* treatment arm followed this illustrated timeline for the duration of study participation. After intervention training and completion of 6 weeks in *MySafeRx* remote recovery coaching check-ins, participants either chose to end study participation or continue coaching check-ins for up to two weeks, a potential total of 8-weeks of study participation. Individuals in the Treatment Arm were expected to provide weekly urine toxicology screens for their initial 9 weeks of study participation, and again at a week 20 study follow-up.

If eligible for the study, participants were randomized to either the study treatment arm to participate in the *MySafeRx* intervention or standard care (SC), the continuation of typical OBOT B/N treatment. Participants were randomized once the study coordinator opened a confidential, sealed envelope with an assigned study number, which had been randomly generated by the study biostatistician. Participants in the *MySafeRx* intervention arm received six weeks of mobile recovery coaching and medication adherence monitoring via the *MySafeRx* program and corresponding mobile Android application (Schuman-Olivier et al., 2018). After completion of 6 weeks in *MySafeRx*, participants were offered an optional two additional weeks of study participation for a gradual taper with reduced frequency of *MySafeRx* support at the conclusion of the initial six weeks, for a potential total of 8-weeks of study intervention (Fig. 1). Seven of nine participants (77%) randomized to the treatment arm who were still enrolled at 6 weeks opted to continue study participation for an additional two weeks.

Participants in the intervention arm attended an in-office technology training on cell phone usage and the *MySafeRx* mobile platform prior to beginning daily video-supervised self-administration of B/N through the *MySafeRx* mobile application platform. This in-office training occurred within one week of participant study randomization. Day 1 of the study for those randomized to the intervention arm was defined as the first day of supervised self-administration of a B/N dose with a *MySafeRx* MRC through the mobile Android *MySafeRx* application. Day 1 of the study for those randomized to the control arm (SC) was defined as the date of study randomization.

#### 2.3.1 *MySafeRx* platform overview

The *MySafeRx* platform provides medication adherence monitoring and mobile recovery coaching support for people with OUD during periods of vulnerability or instability through the facilitation of confidential communication between the patient's clinicians and the trained MRCs who check-in with participants via Zoom videoconference. Four key platform components include: (1) the MedicaSafe 3000 electronic pill dispenser with unique medication release codes transmitted via Android app (MedicaSafe, Inc.); (2) mobile text messaging with programmed alerts reminding participants about upcoming video-conferencing check-ins with MRCs and confidential communication with MRCs to initiate check-ins; (3) daily remote Zoom video-conferencing with a motivational intervention delivered by trained MRCs; and (4) a standardized protocol for supervised self-administration of medication by video. All components are integrated through the *MySafeRx* smartphone application and web portal, as described previously (Schuman-Olivier et al., 2018). Study participants were provided Android mobile phones on an as-needed basis, complete with corresponding mobile data packages for reliable mobile application access from any location. Participants in the intervention study arm were provided an additional non-electronic Master Lock combination lockbox with a 1-day rescue dose of prescribed B/N medication that could be accessed in the case of a dispenser related technology failure.

#### 2.3.2 Mobile recovery coaching, training and certification

All study staff and MRCs were certified adherent in Motivational Interviewing (MI) technique using a standardized patient interview based on MITI4 (Denise Ernst Consulting) (Moyers et al., 2016) prior to coaching *MySafeRx* participants. MRCs came from a variety of professional backgrounds, and were recruited through relevant professional communities after completing an advertised MI training or through networks of people trained in MI. The composition of those who participated in the training and became MRCs varied during the trial (e.g., research coordinators with a bachelor's degree, pre-medicine students, psychiatry residents, recovery coaches, unlicensed clinicians with a master's level in counseling, master's in public health, social work trainees, psychology post-docs, licensed psychologists, and licensed practicing nurse). Several MRCs supported this study for multiple years. Others provided coaching in six-month increments, for a few hours a week during a school year or while they were in clinical training program. Given the various backgrounds and levels of clinical experience that potential coaching candidates had, certification in MI provided the baseline core training for all MRCs. Though many study coordinators trained to be MRCs to support this study if needed for backup coverage, the majority of MRCs were hired after being recruited and trained as described above and paid as hourly contractors. MRCs conducted daily virtual medication adherence and recovery coaching check-ins through the *MySafeRx* mobile application via secure password protected Wi-Fi or broadband connections from their residences. On-call study managers were available 24/7 to support MRCs in case of emergency or technology issues, or instances when participants missed check-ins and needed to access medication outside a scheduled coaching session. Participants had some level of flexibility in selecting a MRC of their choice.

#### 2.3.3 *MySafeRx* mobile app and clinical workflow

The *MySafeRx* Android application provides an encrypted HIPAA-compliant vehicle for text messaging and daily medication adherence monitoring with video embedded using Zoom's application programming interface (API). In this study, participants were required to schedule and join a 30-minute video session with a coach to receive a medication access code each day. MRCs were trained to observe B/N medication placement under the tongue and verify medication dissolution. If a participant was unable to join a videoconference, MRCs were instructed to provide the medication code by phone and to document this issue with a note to the study on-call manager and the participant's clinical team. For participants with a history of missing scheduled check-ins, MRCs were encouraged to focus MI around participants’ program-interfering behaviors to help overcome any feelings of ambivalence and encourage future videoconferencing attendance.

After each video check-in, MRCs completed a Daily Recovery Report through the platform's application interface, documenting medication adherence, patient-reported substance use and daily recovery goals, and relevant additional notes. These reports were visible to the MRC team and delegates assigned to a participant's clinical care team for refence. In the event of a safety concern, homicidal or suicidal ideation, illicit drug use, or consecutive days of medication non-adherence, a participant's care team received an email from the application's rapid alert system advising them to review the details of the event. B/N prescribers, as well as nurse care managers and counselors, were provided the option to access the *MySafeRx* web clinician interface to review coaching notes and reports detailing patient conversation during scheduled daily check-ins.

### 2.4 Assessments

All randomized participants for both arms were asked to complete weekly feasibility, for study weeks 1–6 through the Research Electronic Data Capture (REDCap) web-based application (Harris et al., 2009), while all *MySafeRx* participants were asked also to complete weekly usability and acceptability surveys. Feasibility was assessed as at least two-thirds of study participants self-administering B/N on more than 5 of 7 days per week with no reports of substantial medication diversion. Usability was defined as at least two-thirds of study participants achieving at least 90% competency with the study platform, a standard defined through a cutoff usability score greater than 68 on the validated System Usability Scale (Peres et al., 2013). Acceptability was defined by an overall mean satisfaction score of more than 3 out of 5 on a satisfaction scale at the end of the intervention period. Finally, participants in both arms were expected to provide weekly urine toxicology screens for the first 9 weeks of study duration, and again at week 20 follow-up (Fig. 1).

### 2.5 Participant compensation

All randomized participants were compensated up to $100 in gift cards for study participation. Participants randomized to the *MySafeRx* arm could receive up to $158 in additional compensation for their additional time: $10 for technology training, $30 for time spent refilling their medication dispenser at a local pharmacy, $2 for each daily video encounter completed (up to 42 encounters). This additional compensation was provided after the participant's study phone and medication dispenser were returned undamaged to compensate for increased daily study tasks.

### 2.6 Data analysis

Intervention and SC groups were compared on baseline characteristics using t-tests and chi-square tests for continuous and categorical variables, respectively. Among the intervention group, we compared study outcomes (feasibility, usability, acceptability, and satisfaction of *MySafeRx*) by self-reported sex. Due to small sample size, our analytic approach was primarily descriptive. All analyses were conducted using Stata version 16 (StataCorp, 2019). Results were considered statistically significant at *p*<0.05. Documented anecdotal evidence from MRCs, *MySafeRx* study coordinators, and the PI about medication adherence behaviors, sharing and diversion and were recorded throughout the study duration in MRC notes or as notes to file by study staff. These were reviewed and analyzed for key themes around diversion and adherence to contextualize the quantitative data presented.

## 3 Results

### 3.1 Study referrals

**Fig. 2:**
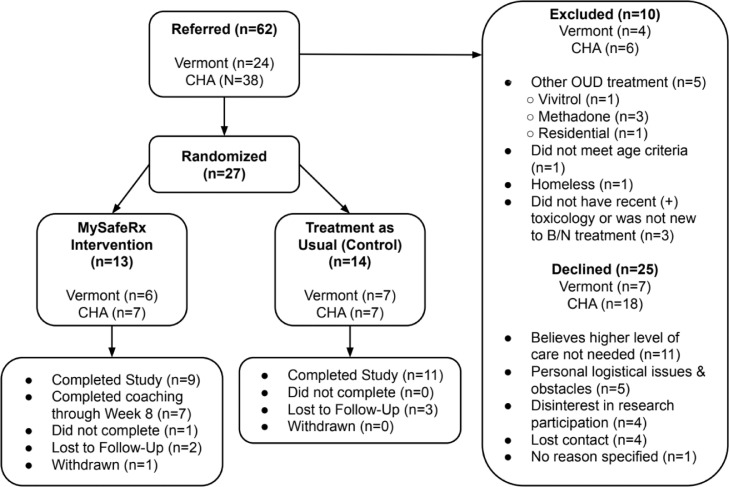
CONSORT diagram. This CONSORT diagram illustrates quantities of individuals referred to the study (*n* = 62), as well as the total number randomized (*n* = 27) and those specifically randomized to the *MySafeRx* Intervention arm (*n* = 13) and the Standard Care (Treatment as Usual/Control) arm (*n* = 14). The total number of referred participants excluded from study participation is also detailed here (*n* = 10). Reasoning behind these exclusions is included here, as well as the total number of individuals who declined study participation after referral at both sites (*n* = 25).

Of 62 referrals, 25 individuals declined study participation (Fig. 2). Reasons for declining study participation among referred individuals can be categorized into five primary themes (Fig. 2): (1) believes higher level of care is not needed (*n*=11); (2) personal logistical issues (*n*=5); (3) disinterest in research participation (*n*= 4); (4) lost contact (*n*=4); (5) no reason specified (*n*=1). People who declined the study generally wished to continue primary care B/N treatment or group-based opioid treatment only (Sokol et al., 2020, 2019, 2018) without additional coaching or daily monitoring of medication adherence. Many people referred by their provider for supervised dosing due to their prescriber's adherence concerns, did not feel they had an issue with medication adherence, nor did they feel a need for supervised dosing. Personal logistical issues included: lack of transportation, work scheduling conflicts, limited private time and space for MRC check-ins, inability to check-in daily, and homelessness. Those disinterested in research participation reported being anxious about study requirements and participation or discomfort with videoconferencing. Several people did not want to take medication all at once during a coaching check-in. In several cases where rationale for not participating was not always clear, medication diversion was often suspected by the clinician or reported later by the referred patient. Those who did not complete the study intake or were discharged before beginning study participation were classified as having been lost to follow-up.

### 3.2 Study participant characteristics

Study participants (*n*=27) were 100% White and English-speaking with an average age of 31.9 years (see Table 1 for additional demographics). Qualifications for study participation included (1) a positive illicit opioid screen in the 30 days prior to study participation (55.5%) or (2) missed opioid toxicology screens with suspected illicit drug use as indicated by the clinical provider. Past use of cocaine was very common and (85.7%) a substantial number of participants had a positive cocaine screening in the 30 days prior to study participation (25.8%). Of the participants randomized to the intervention arm, 15.4% were referred because of the need for a higher level of care (Table 1). Of the participants randomized to the intervention arm, 53.9% were referred due to a positive illicit opioid screen in the 30 days prior to study participation (Table 1). In the intervention arm, 23.1% of participants were referred due to a positive cocaine screening in the 30 days prior to study participation (Table 1). Exploratory efficacy analyses were underpowered and did not show significant differences in urine toxicology results between participants randomized to the standard care arm and those randomized to the intervention arm. Furthermore, as participants randomized to the standard care arm (*n*=14) did not complete the primary aims surveys, detailed results will be discussed here for intervention arm participants (*n*=13).

**Table 1:** Study participant demographics.

Baseline characteristics of *MySafeRx* trial participants (*n* = 27)		
	Control (%)*n* = 14	Intervention (%)*N* = 13
Demographics		
Age (mean/SD)	33.6 (11.4)	30.2 (6.1)
Sex		
Female	8 (64.3%)	9 (66.7%)
Race/Ethnicity		
White	14 (100%)	13 (100%)
Married		
Yes	3 (21.4%)	2 (15.4%)
Employed		
Yes	5 (35.7%)	4 (33.3%)
Education		
Some high school	3 (21.4%)	2 (15.4%)
High school graduate or GED	6 (42.9%)	3 (23.1%)
Some college	3 (21.4%)	5 (38.5%)
College Graduate	2 (14.3%)	2 (15.4%)
Primary referral reason		
Need higher level of care	2 (14.3%)	2 (15.4%)
Positive for illicit opioids	8 (57.1%)	7 (53.9%)
Positive for cocaine	4 (28.6%)	3 (23.1%)
Lifetime substance use		
Heroin	9 (64.3%)	7 (53.9%)
Prescription opioids	12 (85.7%)	9 (69.2%)
Non-prescribed buprenorphine	8 (64.3%)	5 (38.5%)
Fentanyl	8 (64.3%)	5 (38.5%)
Alcohol	12 (85.7%)	11 (84.6%)
Cocaine	12 (85.7%)	10 (76.9%)
Marijuana/hashish	12 (85.7%)	11 (84.6%)
Benzodiazepines	9 (64.3%)	5 (38.5%)
Tobacco	13 (92.9%)	11 (84.6%)
Methamphetamine	2 (14.3%)	1 (7.8%)
Stimulants	6 (42.9%)	7 (53.9%)

Demographics pertaining to study participants randomized to the Standard Care (Control) and Treatment (MySafeRx Intervention) arms are provided. Statistical significance was defined as *p<0.05, **p<0.01, ***p<0.001.

### 3.3 Mobile recovery coaching frequency

Of the 13 participants randomized to the *MySafeRx* intervention group, 12 completed daily remote check-ins with MRCs (Table 2). One participant was withdrawn from the study after randomization, so only demographic information was collected for this individual and he did not meet with MRCs. During the 42-day required study period, men (*n*=4) and women (*n*=8) met with MRCs an average of 32 (SD = 14) days and 47 (SD = 6) respectively (Table 2). At the end of the 42-day required study period, participants were offered the option to check-in with MRCs for an additional 14 days based on participant's clinical need and choice. Of the seven intervention arm participants who met with MRCs during the additional 14 days, women checked in on average more often during the 14-day period than men (Table 2).

**Table 2:** *MySafeRx* platform: major outcomes.

*MySafeRx* platform: major outcomes	Female	Male	All Participants
Feasibility (Week 6)	*n* = 8	*n* = 4	*n* = 12
Adequate Medication Adherence	68.7%	57.9%	64.3%
Usability (Week 6)	*n* = 8	*n* = 4	*n* = 12
Mean System Usability Scores (Adequate Usability Cut-off: a score of 68)	80.2	72.5	78.4
Average number of coaching check-ins	*n* = 8	*n* = 4	*n* = 12
Average Number of Coaching Check-in Days During Study Participation (SD)	47 (6)	32 (14)	42 (15)
Acceptability (Week 6)	*n* = 6	*n* = 3	*n* = 9
*MySafeRx* technology			
The electronic pill dispenser has been easy to use.	4.3 (0.8)	3.7 (1.5)	4.1 (1.0)
The videoconferencing has been easy to use.	4.3 (0.8)	4.0 (1)	4.2 (0.8)
*MySafeRx* coaching			
Checking in with a Mobile Recovery Coach every day has been helpful to my recovery.	4.2 (0.8)	3.3 (2.1)	3.9 (1.4)
I feel like the Mobile Recovery Coaches care about me and have wanted to help me reach my goals.	4.8 (0.4)	3.7 (2.3)	4.4 (1.3)
General program satisfaction			
The *MySafeRx* program has not usually conflicted with other commitments in my daily life.	3.7 (1.5) *	1.3 (0.6)	2.9 (1.7)
The daily video check-in has been supportive during a time when I felt I needed a lot of help.	4.5 (0.8	3.3 (2.1)	4.1 (1.4)
I would recommend *MySafeRx* to a friend starting buprenorphine treatment.	4.7 (0.5)	3.0 (2.0)	4.1 (1.4)
I wish I did not get involved with the *MySafeRx* program.	1.0 (0.0) *	3.0 (2.0)	1.7 (1.4)
The *MySafeRx* program has helped me take my medications on a more regular schedule.	4.5 (0.5)	3.3 (2.1)	4.1 (1.3)
Post-study satisfaction survey results (Week 6)	*n* = 6	*n* = 3	*n* = 9
Technology			
The *MySafeRx* platform was user-friendly.	4.3 (1.0)	3.3 (2.1)	4.0 (1.4)
Coaching			
The daily video check-in visits helped me remain abstinent from drug use.	4.2 (1.0)	3.3 (2.1)	3.9 (1.4)
General program satisfaction			
The *MySafeRx* platform prevented me from selling or giving away my medication	4.0 (1.1)	3.0 (2.0)	3.7 (1.4)
The entire *MySafeRx* medication monitoring program was stressful.	1.7 (0.8) **	4.3 (1.1)	2.5 (1.6)

Feasibility, usability, acceptability, average number of coaching check-ins, and post-study satisfaction survey results based on participant feedback. Acceptability and Post-Study Satisfaction Scales ranges from 1 to 5, with ‘1′ reflecting a response of “Strongly Disagree” and ‘5′, a response of “Strongly Agree.” Sample sizes reflect the number of participants who completed coaching check-ins and surveys. Feasibility, Usability and ‘Average Number of Coaching Check-in’ data were recorded for 12 participants; 1 participant was randomized but was withdrawn from study participation prior to completing these scales. A total of 9 randomized participants completed ‘Acceptability’ scales and the Post-Study Satisfaction Survey. 4 individuals did not: 1 participant was withdrawn from study participation, 2 participants did not complete these scales despite multiple reminders from research coordinators, and 1 Vermont participant was lost to follow-up having not complete these acceptability scales. Significance values are defined here as *p<0.05 and **p<0.01.

### 3.4 Feasibility

MRCs confirmed supervised B/N self-administration at least one time for 12 *MySafeRx* participants with an average percentage of 64.3% of study days with observed doses confirmed (*n*=12) (Table 2), which was less than the anticipated 71% representing 5 out of 7 days of adherence. Men (*n*=4) and women (*n*=8) had confirmed medication adherence on an average of 57.9% and 68.9% of study days, respectively. Per the monitoring surveys, no participant reported medication diversion by selling or sharing their prescribed medication during the study. However, one participant tampered with her dispenser, and another called the MRC after her session and stated that she had only taken one pill with the intent of sharing the other one.

### 3.5 Usability

Participants in the intervention arm assigned the *MySafeRx* intervention a mean System Usability Score of 78.4. (*n*=12) (Table 2). Men (*n*=4) assigned the *MySafeRx* intervention a mean usability score of 72.5 (Table 2). Comparatively, women (*n*=8) assigned the *MySafeRx* intervention a mean usability score of 80.2 (Table 2).

### 3.6 Acceptability

Satisfaction surveys assessed participant endorsement of the *MySafeRx* intervention technology, Mobile Recovery Coaching, and General Program Satisfaction via a scoring scale with 1 corresponding to a response of “Strongly Disagree” and 5 corresponding to a response of “Strongly Agree” (Table 2). The plus and minus notation for the subsequent results denotes standard deviations. On average, participants who completed the acceptability scales (*n*=9) agreed that the electronic pill dispenser was “easy to use” (4.1 ± 1), videoconferencing was “easy to use” (4.2 ± 0.8) and supportive during times when they needed “a lot of help” (4.4± 1.3), and that coaches cared about them and wanted to help them “reach their goals” (4.4 ± 1.3). In addition, participants agreed that the *MySafeRx* intervention helped them “take medication on a more regular schedule (4.1 ± 1.3) and, on average, refuted the statement “I wish I did not get involved with the *MySafeRx* program” (1.7± 1.4). Satisfaction scores for multiple variables showed significant differences in response between men (*n*=3) and women (*n*=6). Women more strongly refuted the statement that they “wished they did not get involved with the *MySafeRx* program,” assigning an average score of (1.0± 0) compared to the average male score of (3.0± 2) (*p*<0.05). Another noted trend indicated that women were more likely to recommend *MySafeRx* to a friend starting buprenorphine treatment (4.7 ± 0.5), compared to the average male score of (3.0 ± 2) (*p*>0.05). Additionally, women assigned a significantly higher score to the survey question assessing whether ‘The *MySafeRx* program has not usually conflicted with other commitments in my daily life” (Table 2). Women indicated they more strongly agreed with that statement, assigning a score of (3.7 ± 1.5) compared to the average male score of (1.3± 0.6) (*p*=0.04).

### 3.7 Post-study satisfaction survey results

Among the participants randomized to the study intervention arm, 9 of 13 individuals completed the Post-Study Satisfaction Survey upon completion of their time in the study; 2 participants did not complete the survey despite reminders from study research coordinators, and no survey data were collected aside from demographic information for the participant withdrawn from the study as well as a Vermont participant who was lost to follow-up. The Post-Study Satisfaction Survey similarly asked questions about the *MySafeRx* platform technology, Mobile Recovery Coaching, and General Program Satisfaction, with a scoring scale of 1 corresponding to a response of “Strongly Disagree’ and 5 corresponding to a response of ‘Strongly Agree’ (Table 2). The plus and minus notation for the subsequent results denotes standard deviations. On average, all respondents agreed that “the *MySafeRx* platform was user-friendly (4.0 ± 1.4), that daily videoconferencing with coaches helped them “remain abstinent from drug use (3.9 ± 1.4), and that the platform prevented the “selling or giving away” of medication (3.7 ± 1.4). A significant difference in scoring between men and women was evident in survey responses agreeing or disagreeing with the statement: “The entire *MySafeRx* medication monitoring program was stressful.” Women more strongly disagreed with that statement, assigning an average score of (1.7 ± 0.8) compared to the average male response score of (2.5 ± 1.6) (*p*<0.01).

### 3.8 Clinical learnings through *MySafeRx* study participation: anecdotal evidence

Anecdotal clinical learnings recorded by *MySafeRx* staff during study recruitment and participation additionally clarified several major themes around the role the platform played in the clinical environment and the impact of the clinical context on *MySafeRx*. These anecdotes illustrate the ways in which *MySafeRx* better informed clinical teams about daily patient activities and treatment engagement including through 1) exposing illicit activity to the clinical team, 2) exposing socioeconomic challenges impacting patient care, and 3) elucidating the pervasiveness of medication sharing, especially among siblings.

In one situation, a patient who previously had not reported illicit activity to clinical staff spoke honestly about his role as a narcotrafficker of Fentanyl. He did so as a result of *MySafeRx* daily videoconference monitoring and his consequent concerns about law enforcement monitoring. During another coaching check-in, a participant further explained her unwillingness to speak with the clinical team by video: her boyfriend ran an illicit cannabis cultivation facility from her home. These situations allowed for deepening of individual clinical treatment, bringing the nature of these risky behaviors, which had been negatively impacting their recovery, into the therapeutic relationship. Several patients expressed financial difficulties and they reported selling their B/N in order to afford their B/N co-pay. Others sold their B/N to purchase other drugs (e.g., cocaine). Two referred patients disclosed domestic violence situations to *MySafeRx* study staff, relaying physical threat and verbal abuse details that had not otherwise been revealed to the clinical team. Finally, a few young adult patients disclosed complex family dynamics involving medication sharing and medication stealing with their siblings, which were circumstances not recognized prior to this level of adherence monitoring. This aligned with prior literature which suggested that SUD among first-degree relatives is a particularly strong risk factor for attrition among young adults (Dayal and Balhara, 2017, Schuman-Olivier et al., 2014).

These *MySafeRx* staff impressions begin to reveal the complexities and challenges of study implementation for high-risk individuals in OUD treatment. Such revelations led clinical team members to recommend *MySafeRx* for unstable patients with OUD, even as patients became increasingly likely to leave the addiction specialty clinic for less restrictive care settings as prescribing standards shifted to include ED prescribing and community prescribing with less intensive toxicology and adherence monitoring overtime.

## 4 Discussion

### 4.1 The impact of *MySafeRx* usability, participant acceptability, and platform satisfaction

These results provide a better understanding of the feasibility, usability, and acceptability of the *MySafeRx* platform. Acceptability of the *MySafeRx* platform and mobile recovery coaching was high among participants randomized to the treatment arm. These results show that despite variations in the number of total coaching check-ins among participants in the treatment arm, both men and women indicated high acceptance of the interacting *MySafeRx* technical components with especially high levels of acceptability of mobile recovery coaching. Yet, the study results presented here suggest that women, on average, were more likely to find *MySafeRx* supportive during periods of instability (Table 2). Women also found the routine of checking in with a MRC every day to take medication less conflicting with their daily schedules and other life commitments compared with men. These results are consistent with previous literature analyzing the acceptability of technology interventions for substance use disorder among women (Campbell et al., 2015; Loree et al., 2019; Stinson et al., 2020; Sugarman et al., 2020). Increased access to technology intervention platforms for remote treatment of substance use disorders (SUDs) has historically been more widely accepted by women (Loree et al., 2019; Sugarman et al., 2020). Additionally, remote technology interventions support gender specific treatment for SUDs, allowing women to access personalized MI coaching support for the co-occurring mental health disorders, trauma and abuse they are more likely to want to address while in addiction treatment (Sugarman et al., 2020). Given the limited sample size of this study, future research should further examine and confirm these results to verify the discrepancies in response to participant satisfaction with the *MySafeRx* intervention between women and men utilizing the platform.

### 4.2 The implementation of *MySafeRx* within a changing B/N prescribing landscape

Nearly half of individuals (44%) referred to the study declined participation due to their perceived lack of need for adherence monitoring or coaching despite clinician referral. The adherence monitoring aspect of the intervention and especially the locked electronic pill dispenser did not have broad appeal, and its acceptance was found to be extremely context dependent. Across the nation in the past 5 years, an expansion of B/N prescribing has led to increased B/N access and lowered barriers to initiating care, representing a major policy success over the past decade, engaging more people in OUD treatment (Lane et al., 2021; Stinson et al., 2020). With this success, B/N maintenance treatment became readily available in the communities where the study took place with fewer expectations from providers for toxicology testing and adherence monitoring. As accessibility increased, the level of interest in the intervention dropped off precipitously with the change in context and contingencies. Once patients were started in care with limited levels of monitoring, then the suggestion of a higher level of care by clinicians was often experienced as punishment; whereas the same intervention suggested at the outset of care was often strongly welcomed by patients.

Early on in the treatment development project, *MySafeRx* was heartily welcomed by patients since it was often presented at the time by local clinicians as one option to avoid referral to methadone. Later, when adherence concerns were raised by staff and staff presented *MySafeRx* as one potential alternative to methadone, patients felt they had many other treatment options. As a result, they more frequently declined study referral despite clinician recommendations or chose to leave the addiction specialty clinic in favor of a community program with lower levels of toxicology testing and medication adherence monitoring. Study staff noted these themes specifically when two participants chose external vivitrol and methadone treatment over *MySafeRx* participation, both instances due to personal aversion to regimented adherence monitoring. This experience suggests that the feasibility of the *MySafeRx* intervention is dependent on context and contingencies and does not have broad appeal in contexts where B/N is readily available in the community with lower levels of monitoring. The majority of patients with OUD struggling in opioid treatment may seek treatment support in settings with less monitoring instead of seeking treatment options with more adherence monitoring, even when considering the convenience of taking medication via digital lockbox and checking in with recovery coaches from home.

The demand for adherence-focused interventions seemingly diminished as the context changed. While recruitment was robust in the proof-of-concept study conducted prior to this RCT and during the initial months of the RCT, recruitment fell in parallel with the rise in community emergency department prescribing at both sites. The study team attempted to increase outreach to local emergency departments. However, referrals from the ED were extremely rare. Many primary care providers (PCPs) simultaneously reduced traditional expectations for adherence (i.e., pill counts, toxicology monitoring), and then increasingly struggled to refer patients to the study who they felt may benefit from more support or greater adherence monitoring. Community prescribers in these regions became more comfortable with the potential of prescribing in the face of partial and possible non-adherence and seemed less worried about the negative impacts of B/N diversion. This noted trend is congruent with a previously demonstrated difference that greater exposure to and experience with prescribing B/N is associated with lower levels of concern about diversion (Schuman-Olivier at al., 2013). While this may be appropriate and a worthwhile risk to increase access, the impact of context and presence or lack of contingencies in the community on patient perception of *MySafeRx* was remarkable.

The majority of patients in need of B/N for OUD can now access a prescription regardless of their geographic location in the U.S. (Li et al., 2016; Lin and Knudsen, 2019; Marino et al., 2019; Paulsen et al., 2020; Skolnick, 2018; Zoorob et al., 2018). Given this increase in B/N prescribing in the years leading up to the COVID-19 pandemic, and further lifting of B/N prescription restrictions due to the COVID-19 pandemic (Uscher-Pines et al., 2020), it has become increasingly difficult to establish a high level of adherence monitoring for patients prescribed B/N. Consequently, any question of the utility of an adherence monitoring program may become moot in many community contexts (Krawczyk et al., 2020), especially during COVID-19. However, as this paper outlines, important lessons have been learned that may help inform how remote adherence monitoring for high-risk patients may be delivered successfully in certain community contexts, especially in states and regions where there is still strong concern by providers or family members about B/N diversion or non-adherence (Sharp et al., 2021).

### 4.3 Limitations

There are several limitations to this study. The small sample size made it difficult to make definitive inferences about the effectiveness of the intervention, and intervention preferences between male and females, as well as to discern whether trends discussed here would persist among a larger sample population. Furthermore, although this limited study sample reflects the demographics among the recruitment populations in Bennington, VT and Somerville, MA, the sample lacks diversity as all participants who were randomized and remained in the study were non-Hispanic White. Results therefore may not be generalizable nor currently relevant for populations who disproportionately face barriers to substance use disorder treatment (Stinson et al., 2020). This study was also impacted by several unexpected events including the sudden death of the primary research coordinator and the unanticipated shutdown of one of the main OUD treatment programs in Vermont. These challenges resulted in a consolidation of recruitment efforts to Somerville, MA mid-way through the study. Active study recruitment ended in the greater Boston metro area when the healthcare system closed in person research due to COVID-19 precautions in March 2020.

From a study design standpoint, another limitation involves study participant compensation. As noted above, participants randomized to the intervention arm received additional compensation for the additional amount of time spent interacting with study staff compared with those randomized to standard care. Furthermore, although urine toxicology was collected for participants randomized to the treatment arm for study Weeks 1–9 to monitor drug use and validate self-reported medication adherence in the survey data, toxicology data is not reported here due to a substantial amount of missing data from the majority of our Vermont participants, whose results were unable to be accessed after our staff member's death. In addition, three individuals randomized were either withdrawn from the study, pursued alternate treatment after randomization, and/or did not give consent for the study team to collect urine toxicology data from a second treatment facility.

### 4.4 Future studies

Future studies should examine the differential impacts of individual components of the multi-component adherence intervention (Steinkamp et al., 2019). For example, electronic pill dispensers were required for diversion prevention in the study design. However, this added complexity to the intervention and led to numerous potential referrals declining participation. Future studies that offer more flexibility around the timing of electronic pill dispensing may enhance study enrollment and engagement. Furthermore, since participants were most satisfied with the compassionate attitude of MRCs, it could be helpful to isolate the role that recovery coaching plays in enhancing participant engagement when electronic pill dispensing is not required from the intervention outset. These ideas could realistically be incorporated into a large-scale RCT to further assess the mobile platform components and coaching elements of *MySafeRx*, including why women in this current study found *MySafeRx* more feasible, acceptable, and satisfactory than men.

## 5 Conclusions

This study has provided critical insights into the need for recovery coaching support during periods of instability, particularly for women. We encountered difficulties recruiting study participants due to the changing B/N prescribing landscape that has lowered medication access barriers for those seeking OUD treatment. The study intervention with daily adherence monitoring and locked electronic pill dispensers was less attractive to patients who were referred because they were inconsistently adhering to outpatient OUD treatment requirements for medication adherence. Still, coaching reports and study staff impressions recorded during the study enrollment period alerted site clinical staff to important treatment issues, which allowed for timely modifications to patient treatment when needed. Finally, the knowledge generated from coaching reports, participant feedback, and staff impressions will also be useful reference for future telemedicine OUD implementation projects and research.

## Authors' contribution

Funding Acquisition: Zev Schuman-Olivier, MD, Jacob Borodovsky, PhD, Lisa A. Marsch PhD

Conceptualization: Zev Schuman-Olivier, MD, Jacob Borodovsky, PhD, Lisa A. Marsch PhD, Jackson Steinkamp, MD

Formal analysis: Michael Flores, PhD

Software: Grace Janzow, BA, Jackson Steinkamp, MD (primary application developer)

Project Administration: Cassandra Harding, MPH, Grace Janzow, BA

Original Draft Writing: Grace Janzow, BA, Cassandra Harding, MPH, Jacob Borodovsky, PhD, Zev Schuman-Olivier, MD

Review and Editing: Grace Janzow, BA, Cassandra Harding, MPH, Jacob Borodovsky, PhD, Zev Schuman-Olivier, MD, Lisa A. Marsch, PhD

## Author disclosures

This work was supported by a treatment development grant from NIDA (R34DA040086, PI: Schuman-Olivier), a center grant (P30DA029926, PI: Marsch) and philanthropic grants from the Sandra and Arnold Gold Humanism Research Fund (Schuman-Olivier) and Russell Berrie Foundation (Schuman-Olivier).

## Declaration of Competing Interest

There are no conflicts for authors to disclose at this time. *MySafeRx* Inc. is a non-profit organization with a mission to support the development, implementation, and dissemination of adherence-based solutions for substance use and mental health treatment. Drs. Schuman-Olivier, Borodovsky, and Marsch are members of the board of directors and officers for *MySafeRx.* Ms. Harding is also an officer for *MySafeRx*. Upon funding of the NIDA grant and initiation of the project, Geisel School of Medicine at Dartmouth conducted a review of the project and concluded there was no financial conflict of interest.

## Appendix Supplementary materials

Image, application 1
